# Characteristics of SARS-CoV-2-associated severe episodes of monoclonal gammopathy-associated capillary leak syndrome (Clarkson disease)

**DOI:** 10.1186/s13613-025-01483-7

**Published:** 2025-05-26

**Authors:** Nissim Grinberg, Maddalena Alessandra Wu, Quentin Moyon, Sybille Merceron, Yannick Fedun, Marie Gousseff, Romain Sonneville, François Lhote, Elie Azoulay, Jean-Herlé Raphalen, David Saadoun, Ygal Benhamou, Jean-Paul Mira, Guillaume Dumas, Pierre Bay, Jérôme Devaquet, Laurent Argaud, Marc Lambert, Avinash Aujayeb, Basile Henriot, Amandine Bichon, Thomas Bocar, John Harty, Remo Melchio, Franck Leibinger, Laure Calvet, Tomas Urbina, Laurent Bodson, Jean-Marie Tonnelier, Danielle Reuter, Emmanuel Canet, Gilles Blaison, Julien Maizel, Nicholas Sedillot, Laurence Dangers, Vincent Eble, Franco Verlicchi, Stanislas Faguer, Jonathan Montomoli, Geoffroy Dingemans, Marc Mikulski, Jonas Pochard, Fabrice Uhel, Fleur Cohen-Aubart, Charles-Edouard Luyt, Alexis Mathian, Alain Combes, Riccardo Colombo, Zahir Amoura, Marc Pineton de Chambrun, the EurêClark Study Group

**Affiliations:** 1 Service de Médecine Intensive-Réanimation, Sorbonne Université, Assistance Publique-Hôpitaux de Paris (AP-HP), Hôpital La Pitié–Salpêtrière, 47–83, Boulevard de l’Hôpital, 75651 Paris Cedex, France; 2 Division of Internal Medicine, Department of Biomedical and Clinical Sciences, ASST Fatebenefratelli Sacco, Luigi Sacco Hospital, University of Milan, Milan, Italy; 3 Service de Médecine Interne 2, Centre de Référence National Lupus Systémique, Syndrome des Anticorps Anti-Phospholipides et Autres Maladies Auto-Immunes Systémiques Rares, Sorbonne Université, AP-HP, Hôpital La Pitié–Salpêtrière, Institut E3M, Paris, France; 4 Institut de Cardiométabolisme et Nutrition (ICAN), Sorbonne Université, INSERM, UMRS_1166-ICAN, Paris, France; 5 Service de Médecine-Intensive Réanimation, Hôpital André Mignot, Le Chesnay, France; 6 Service de Réanimation Polyvalente, CH Bretagne Atlantique, Vannes, France; 7 Service de Médecine Interne, CH Bretagne Atlantique, Vannes, France; 8 Service de Médecine-Intensive Réanimation, CHU Bichat, AP-HP, Paris, France; 9 Service de Médecine Interne, Hôpital Delafontaine, Saint-Denis, France; 10 Service de Médecine Intensive-Réanimation, AP-HP, Hôpital Saint-Louis, Paris, France; 11 Service de Réanimation Polyvalente, AP-HP, Hôpital Necker, Paris, France; 12 Department of Internal Medicine and Clinical Immunology, Sorbonne Universités, AP-HP, Groupe Hospitalier Pitié-Salpêtrière, Centre National de Références Maladies Autoimmunes et Systémiques Rares, Centre National de Références Maladies Autoinflammatoires Rares et Amylose Inflammatoire, and INSERM, UMR S 959, Immunology- Immunopathology-Immunotherapy (I3), Paris, France; 13 Service de Médecine Interne, Normandie Université, UNIROUEN, 76031 Rouen, France; 14 Service de Médecine Intensive-Réanimation, AP-HP, CHU Cochin, Paris, France; 15 Service de Réanimation Médicale, CHU Grenoble Alpes, La Tronche, France; 16 Service de Réanimation Médicale, CHU Henri-Mondor, AP-HP, Créteil, France; 17 Service de Réanimation Polyvalente, CH Foch, Suresnes, France; 18 Service de Médecine Intensive-Réanimation, CHU Edouard-Herriot, Lyon, France; 19 Département de Médecine Interne et d’Immunologie Clinique, Univ. Lille, CHU Lille, 59000 Lille, France; 20 Northumbria Specialist Emergency Care Hospital, Newcastle, England; 21 Service de Médecine Interne, GHT d’Armor, Saint-Brieuc, France; 22 Service de Médecine Intensive-Réanimation, Assistance Publique Hôpitaux de Marseille, Hôpital La TimoneMarseille, France; 23 Service d’Accueil des Urgences, CHU Pontchaillou, Rennes, France; 24 Southern Health and Social Care Trust, Craigavon, Northern Ireland; 25 Divisione Di Medicina Interna, A.O. S. Croce E Carle, Cuneo, Italy; 26 Service de Réanimation Polyvalente, CH de Perpignan, Perpignan, France; 27 Service de Médecine Intensive-Réanimation, CHU Gabriel-Montpied, Clermont-Ferrand, France; 28 Service de Médecine Intensive-Réanimation, Hôpital Saint-Antoine, AP-HP, Paris, France; 29 Service de Réanimation, Clinique Saint Gatien Alliance, Saint-Cyr-Sur-Loire, France; 30 Service de Médecine Intensive-Réanimation, CHU de Brest, Brest, France; 31 Service de Médecine Intensive-Réanimation, Centre Hospitalier Sud Francilien, Corbeil-Essonnes, France; 32 Service de Médecine Intensive-Réanimation, CHU de Nantes, Nantes, France; 33 Service de Médecine Interne, Hôpital de Colmar, Colmar, France; 34 Service de Médecine Intensive-Réanimation, CHU d’Amiens, Amiens, France; 35 Service de Médecine Intensive-Réanimation, CH de Bourg-en-Bresse, Bourg-en-Bresse, France; 36 Service de Médecine Intensive-Réanimation, CHU de La Réunion, Saint-Denis de La Réunion, France; 37 Service de Médecine Interne, CH d’Evreux, Evreux, France; 38 Transfusion Medicine Faenza-Lugo, Transfusion Service Ravenna, AUSL Romagna, Ravenna, Italy; 39 Département de Néphrologie et Transplantation D’organes, Centre de Référence des Maladies Rénales Rares, INSERM U1297 (I2MC, Équipe 12), CHU de Toulouse, France; 40 Department of Intensive Care, Ospedale “Infermi”, Rimini, Romagna Local Health Authority Italy; 41 Service de Médecine Intensive-Réanimation, CHU de Nîmes, Nîmes, France; 42 Service de Réanimation Polyvalente, CHT Gaston-Bourret, Nouméa, France; 43 Service de Réanimation Chirurgicale, Hôpital de Bicètre, AP-HP, Le Kremlin-Bicêtre, France; 44 Médecine Intensive Réanimation, AP-HP, Hôpital Louis Mourier, DMU ESPRIT, 92700 Colombes, France; 45 Inserm, Centre d’Immunologie et des Maladies Infectieuses (CIMI-Paris), Sorbonne Université, Paris, France; 46 Division of Anesthesiology and Intensive Care, ASST Fatebenefratelli Sacco, Luigi Sacco Hospital-Polo Universitario-University of Milan, Milan, Italy

**Keywords:** Monoclonal gammopathy-associated capillary-leak syndrome, Monoclonal gammopathy, Clarkson disease, SARS-CoV-2, Intensive care unit

## Abstract

**Background:**

Monoclonal gammopathy-associated capillary leak syndrome (MG-CLS) is a rare condition characterized by recurrent episodes of hypovolemic shock caused by a sudden increase in capillary permeability. The COVID-19 pandemic has been associated with a rise in MG-CLS episodes and increased mortality. We aimed to explore the association between MG-CLS and SARS-CoV-2 infection. We conducted a multicenter retrospective observational study involving MG-CLS patients who were admitted to the intensive care unit (ICU). The primary endpoint was 28-day mortality according to whether SARS-CoV-2 was identified as a trigger.

**Results:**

The study included 84 patients (44% women) with a median age of 55 years [IQR 46–62], accounting for 127 ICU admissions. Most patients (88%) had monoclonal gammopathy, predominantly with an IgG heavy chain (98%). A trigger was identified in 63% of cases, primarily suspected or confirmed viral infections, including 26 episodes of SARS-CoV-2 infection. Within 28 days of ICU admission, 32% of patients died. Episodes triggered by SARS-CoV-2 were associated with a higher need for mechanical ventilation (69% vs. 38%, p = 0.004), renal replacement therapy (54% vs. 31%, p = 0.03), and increased 28-day mortality (42% vs. 17%, p = 0.005). Multivariable analysis revealed that SARS-CoV-2 infection was independently associated with 28-day mortality (OR 4.67 [1.08–20.1], p = 0.04). The use of intravenous immunoglobulins did not improve 28-day survival.

**Conclusion:**

In this large cohort of MG-CLS episodes requiring ICU admission, SARS-CoV-2as a trigger was associated with significantly higher 28-day mortality compared to other triggers. Further research is essential to elucidate the specific mechanisms by which SARS-CoV-2 impacts MG-CLS patients.

**Graphical abstract:**

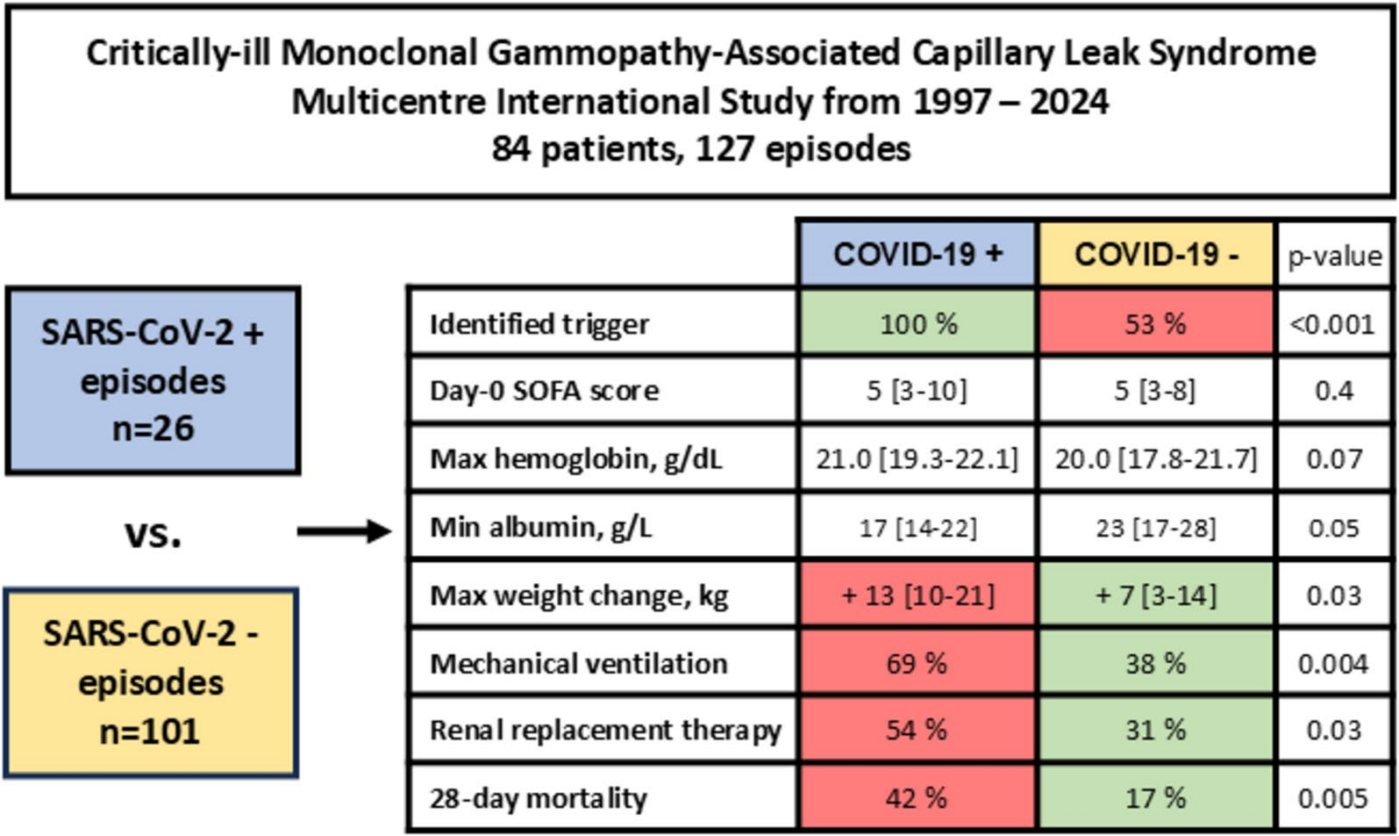

**Supplementary Information:**

The online version contains supplementary material available at 10.1186/s13613-025-01483-7.

## Introduction

Monoclonal gammopathy-associated capillary leak syndrome (MG-CLS), also known as Clarkson disease, is a rare condition characterized by recurrent episodes of hypovolemic shock caused by a sudden increase in capillary permeability [1]. The clinical picture is archetypical, characterized by muscle pain, hypovolemic shock, markedly elevated hematocrit, and low proteinemia. Severe episodes are frequently occur few days after a viral infections. These episodes often require admission to the intensive care unit (ICU) and are associated with significant morbidity (especially compartment syndrome) and mortality [2]. While its pathophysiology remains unknown, Clarkson disease is recognized as a monoclonal gammopathy of clinical significance [3]. No specific treatment has proven effective for managing acute capillary leak episodes [4]. Long-term therapy with intravenous immunoglobulins (IVIG) is the cornerstone for preventing relapses and has been shown to improve long-term survival [5]. The COVID-19 pandemic has been associated with a notable increase in MG-CLS episodes, involving both new and relapsing patients [6, 7]. SARS-CoV-2 infection, as well as its vaccine, has been identified as potential triggers for Clarkson disease recurrence [8]. Following the pandemic, univariate analysis suggested that SARS-CoV-2 infection was associated with increased long-term mortality [9]. It is unclear whether the increased severity of SARS-CoV-2-induced Clarkson syndrome episodes arises from a more severe capillary leak phenotype or the presence of acute respiratory distress syndrome. To explore this further, we aimed to compare the mortality of severe MG-CLS episodes admitted to the ICU based on whether SARS-CoV-2 was identified as the triggering factor.

## Methods

### The European Clarkson’s disease (EurêClark) registry and ethical consideration

As previously reported [10, 11], the EurêClark registry is an international study group that collects Clarkson disease observations and prospectively monitors episodes, preventive treatments, complications and patient outcomes. There is no international consensus on the definition of Clarkson disease or the criteria for its episodes. Established in 1997, this registry received approval from local review boards (AP–HP no.14) and the Commission Nationale de l’Informatique et des Libertés (no. 1001704). All patients, or their legal representatives, provided informed consent for inclusion in the EurêClark database.

### Study design and primary endpoint

We conducted a multicentric retrospective observational study to compare the 28-day mortality of severe MG-CLS episodes requiring ICU admission stratified by the presence of SARS-CoV-2 as a trigger.

### EurêClark and study inclusion criteria

MG-CLS patients were included from January 1, 1997, and monitored until February 29, 2024. Patients could be included even if their first episode occurred before the start date for inclusion. The diagnostic criteria for MG-CLS were as follows: the presence of a monoclonal gammopathy, or a clear justification for its absence (e.g., the patient died before the gammopathy could be investigated or identified during the early phase of the disease); and one or more episodes meeting all of the following criteria [11]: (1) clinical signs of acute hypovolemia and acute edema; (2) hemoconcentration defined as elevated hematocrit or hemoglobin exceeding normal values for age and sex or an increase of > 20% compared to the patient’s last reference value, combined with paradoxical hypoproteinemia; (3) exclusion of alternative causes of secondary capillary leak syndrome or hypoproteinemia [4, 12, 13]. Pediatric patients were not considered for inclusion because none had monoclonal gammopathy [14].

We included every MG-CLS episodes from the EurêClark registry requiring ICU admission. Data for each episode were retrospectively collected by contacting the ICU physicians who managed the cases.

### Data collection and statistical analyses

Standardized forms were used to collect data on epidemiological parameters, clinical manifestations, laboratory findings, ICU treatments, complications and outcomes. The vasoactive score was calculated as follow: dobutamine (µg/kg/min) + 10 × milrinone (µg/kg/min) + dopamine (µg/kg/min) + 100 × epinephrine (mcg/kg/min) + 100 × norepinephrine (µg/kg/min) + 10,000 × vasopressin (µg/kg/min). Following a descriptive analysis of the 127 episodes, only the most recent flare for each patient (n = 84) was included to compare characteristics and outcomes based on COVID status. Continuous variables were reported as medians with interquartile ranges (IQR) and compared using the Wilcoxon Rank-Sum Test. Categorical variables were presented as counts and percentages and compared using the Chi-Squared Test, except when expected cell counts were less than five, in which case Fisher’s Exact Test was used. A COVID-19-related episode was defined by a positive SARS-CoV-2 polymerase chain reaction (PCR) test from any respiratory sample.

All variables were compared based on SARS-CoV-2 status and included in univariable analysis to test their association with 28-day mortality. The multivariable model was designed to evaluate the relationship between SARS-CoV-2 status and 28-day mortality. Predictors such as severity scores, weight changes due to flare, volume of fluid administered, and biological parameters were excluded from the model, as they were considered mediators rather than confounders. We explored collinearity using a correlation matrix and by calculating variance inflation factors (VIF), excluding variables with a VIF ≥ 2. Among the remaining variables, those with a p-value < 0.2 in the univariable analysis were retained for the multivariable model. To account for the correlation between multiple flares in the same patient, we employed a mixed-effects model, incorporating the patient identifier as a random effect and other confounders as fixed effects. Statistical significance was set at p < 0.05. All analyses were conducted using R version 4.2.1.

### Multiple imputation for missing data

To address missing data, multiple imputation was performed on key variables, including associated diseases, clinical scores, clinical characteristics, organ failure indicators, biological parameters, and echocardiographic measurements. The Predictive Mean Matching (PMM) method was applied using the *mice* package in R. This approach imputes missing values by matching them with observed values that are similar based on their predicted values. A random seed was utilized to ensure reproducibility of the imputation process. The imputed values were then integrated into the original dataset, replacing the missing entries to improve data completeness and reliability for subsequent regression analyses. In line with recent recommendations, we did not apply a strict missingness threshold to guide imputation decisions. Instead, we considered both the proportion of missing data and the potential informativeness of each variable, focusing on maintaining model validity and avoiding bias. Only one variable (ICU admission—diuretics start delay, days) had more than 40% missing values, which we decided to exclude from multivariable analysis due to limited auxiliary information to support reliable imputation. All other variables had acceptable missingness and were included in the imputation model. A full summary of variable-level missingness (stratified by COVID status) is provided in Supplementary Table 1.

### Ethical consideration

This study was conducted in accordance with the declaration of Helsinki and utilized the database registered at the Commission Nationale de l’Informatique et des Libertés (CNIL, registration no. 1950673). In agreement with the ethical standards of our hospital’s institutional review board, the Committee for the Protection of Human Subjects, and French law, written informed consent was not needed for the analysis of demographic, physiological and hospital-outcome data, as this observational study did not modify existing diagnostic or therapeutic strategies. However, patients and/or their relatives were informed of their anonymous inclusion in the study.

## Results

### General characteristics of the study population

**Fig. 1:**
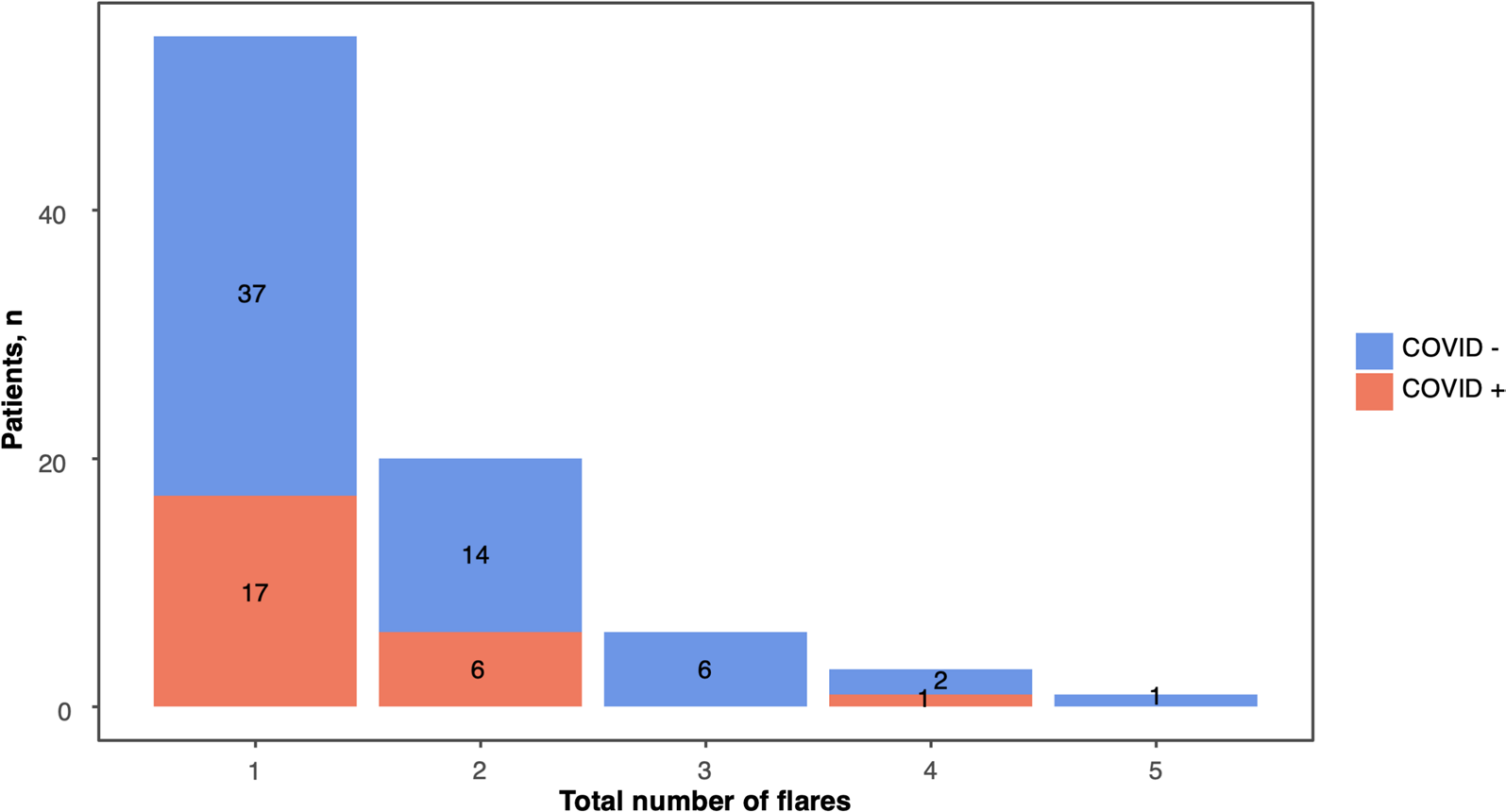
Total number of flares per patient stratified by SARS-CoV-2 status. COVID-19, coronavirus disease 2019

The study included 84 patients, 37 of whom were women (44%), with a median age of 55 years [IQR 46–62] (Table 1). Patients were recruited in 5 countries (France n = 81, Italy n = 36, French overseas territories n = 6, United Kingdom n = 2, Switzerland n = 2) and 63 centers. These patients experienced a total of 127 ICU admission for MG-CLS episodes during the study period. The majority (88%) had monoclonal gammopathy, with 98% exhibiting an IgG heavy chain and 63% a kappa light chain. Recurrent episodes were common, with 39 patients (46%) experiencing at least two episodes, and a median of 1 flare per patient [IQR 1–2]. The distribution of flares per patient is depicted in Fig. 1. Twenty-seven patients (32%) died within 28 days of ICU admission.

**Table 1:** General characteristics of the study population

Variables	n^a^	Every single patient n = 84
Age, years	84	55 [46–62]
Female	84	37 (44)
Monoclonal gammopathy	84	74 (88)
Heavy chain	64	
IgA		1 (1)
IgG		62 (98)
IgM		1 (1)
Light chain	59	
Kappa		37 (63)
Lambda		22 (37)
Number of flares ≥ 2	84	29 (46)
Number of flare	84	1 [1–2]
Day-28 mortality	84	27 (32)

Ig, immunoglobulin. Continuous variables are expressed as mean (standard deviation) or median [interquartile range 25–75]; categorical variables are expressed as n (%).^a^Number of data available

### Description of the 127 episodes: characteristics, treatments and outcomes

The primary reasons for ICU admission were hypovolemic shock (83%), cardiogenic shock (7%), coma (3%), respiratory distress (3%), cardiac arrest (2%) and acute kidney insufficiency (1%, Table 2). A trigger was identified in 63% of cases, predominantly suspected (55%) or confirmed (45%) viral infection, including SARS-CoV-2 in 26 episodes. SARS-CoV-2 infection was most often diagnosed at the time of ICU admission. The median time between a positive PCR test and the onset of MG-CLS symptoms was 1 day [IQR 1–2.75]. Only one episode occurred during the first COVID-19 wave. In the subsequent waves, 15 out of 25 patients had received at least one dose of the vaccine. The values of the WHO ordinal scale and inflammation risk categories were as follows: 9 (16/26, 61%), 5 (4/26, 15%), 4 (4/26, 15%), 8 (1/26, 4%) and 7 (1/26, 4%). The median Sequential Organ Failure Assessment (SOFA) score at ICU admission was 5 [IQR 3–9]. Patients exhibited a median weight gain of 2 kg [IQR 0–8] at ICU admission compared to their previously documented usual weight, with a maximal weight gain in the ICU of 8 kg [IQR 3–18]. Median hemoglobin level at ICU admission was 20.1 g/dL [IQR 18.0–21.9], with a median maximal drop of 9.5 g/dL [IQR 6.3–13.4] during the ICU stay. The median protidemia at ICU admission was 49 g/L [IQR 38–60], while the median maximal creatine phosphokinase (CPK) level in the ICU reached 1329 UI/L [IQR 146–18,173]. The median cumulative fluid therapy administered on day 1 was 4.0 L [IQR 2.1–6.8], with 9% of cases receiving more than 10 L. Mechanical ventilation and renal replacement therapy were required in 44% and 35% of episodes, respectively. The median vasoactive-inotropic score was 118 [IQR 30–313]. IVIG were administered in 32% of flares. The most common ICU complications were compartment syndrome (26%), left ventricular dysfunction (26%), pulmonary edema (17%), arrhythmias or conduction disorders (13%), and acute respiratory distress syndrome (7%).

**Table 2:** Characteristics, treatments and outcomes of all MG-CLS episodes with comparison according SARS-CoV-2 status

Variables	n^a^	All episodes n = 127	SARS-CoV-2 + n = 26	SARS-CoV-2- n = 101	*p-*value
BMI, kg/m^2^	91	25.4 [23.0–28.4]	25.5 [23.2–29.8]	25.4 [23.0–28.2]	0.7
Weight, kg	97	80 [67–87]	72 [63–90]	82 [70–87]	0.6
Cause of ICU admission	127				0.5
Hypovolemic shock		106 (83)	24 (92)	82 (81)	
Cardiogenic shock		9 (7)	0 (0)	9 (9)	
Coma		4 (3)	1 (4)	3 (3)	
Respiratory distress		4 (3)	0 (0)	4 (4)	
Cardiac arrest		3 (2)	1 (4)	2 (2)	
Acute kidney insufficiency		1 (1)	0 (0)	1 (1)	
Identified trigger	127	80 (63)	26 (100)	54 (53)	< 0.001
Type of trigger	127				< 0.001
Confirmed infection		34 (27)	26 (100)	8 (8)	
Suspected infection		42 (33)	0 (0)	42 (42)	
Drug induced		3 (2)	0 (0)	3 (3)	
Hormonal		2 (2)	0 (0)	2 (2)	
None		46 (36)	0 (0)	46 (46)	
Clinical and laboratory findings at ICU admission					
SOFA score at ICU admission	116	53 [3–9]	53 [3–10]	53 [3–8]	0.4
Circulatory SOFA score ≥ 3	118	54 (46)	45 (47)	9 (41)	0.6
Renal SOFA score ≥ 3	118	37 (31)	29 (30)	8 (36)	0.6
Respiratory SOFA score ≥ 3	116	17 (15)	10 (11)	7 (32)	0.02
Liver SOFA score ≥ 3	118	0 (0)	0 (0)	0 (0)	
Neurological SOFA score ≥ 3	118	19 (16)	14 (15)	5 (23)	0.3
Hematological SOFA score ≥ 3	118	5 (4)	4 (4)	1 (4)	> 0.99
APACHE score at ICU	83	19 [14–26]	22 [18–36]	19 [14–25]	0.1
Weight at ICU admission, kg	92	83 [73–92]	81 [72–98]	84 [74–91]	0.9
Weight change at ICU admission, kg	83	2 [0–8]	8 [2–9]	1 [0–7]	0.1
Maximum weight change in ICU, kg	79	8 [3–18]	13 [10–20]	7 [3–14]	0.03
Hemoglobin level at ICU admission, g/dL	125	20.1 [18.0–21.9]	21.0 [19.3–22.1]	20.0 [17.8–21.7]	0.07
Maximal change in hemoglobin in ICU, g/dL	104	9.5 [6.3–13.4]	10.2 [6.2–11.8)	9.5 [6.5–13.5]	0.7
Protidemia at ICU admission, g/L	94	49 [38–60]	47 [41–66]	50 [37–59]	0.7
Minimal protidemia in ICU, g/L	84	39 [29–50]	41 [21–48]	39 [29–50]	0.5
Albumin at ICU admission, g/L	87	25 [18–29]	23 [15–31]	26 [18–29]	0.6
Minimal albumin in ICU, g/L	80	23 [16–27]	17 [14–21]	23 [17–27]	0.05
Creatinine level at ICU admission, µmol/L	124	170 [114–219]	155 [111–213]	175 [116–219]	0.6
Maximal creatinine in ICU, µmol/L	110	219 [149–299]	211 [149–266]	221 [151–299]	0.8
CPK level at ICU admission, UI/L	87	221 [107–1,240]	1,116 [266–2,547]	156 [100–990]	0.002
Maximal CPK in ICU, UI/L	86	1,329 [146–18,173]	8,524 [1,750–11,844]	841 [124–22,624]	0.1
Arterial lactate level at ICU admission, mmol/L	94	4.4 [2.4–7.3]	6.8 [3.4–9.8]	4.1 [2.4–6.7]	0.1
Maximal lactate level in ICU, mmol	85	5.9 [3.3–10.0]	10.0 [7.8–13.3]	5.0 [3.0–8.4]	0.004
Bicarbonate level at ICU admission, mmol/L	102	13.0 [9.8–17.9]	12.0 [8.0–16.0]	13.0 [10.6–18.0]	0.2
In-ICU treatments					
Total fluid therapy^b^					
Fluid therapy on day 1, L	78	4.0 [2.1–6.8]	4.3 [2.0–7.6]	3.6 [2.2–6.5]	0.7
Fluid therapy > 10L on day 1	78	7 (9)	2 (11)	5 (8)	0.7
Mechanical ventilation	124	55 (44)	18 (69)	37 (38)	0.004
Time on mechanical ventilation, days	48	6 [2–10]	5 [3–10]	7 [1–9]	0.9
Renal replacement therapy	127	45 (35)	14 (54)	31 (31)	0.03
Time on renal replacement therapy, days	42	5 [1–16]	2 [1–8]	6 [2–16]	0.2
Vasoactive-inotropic score^c^	43	118 [30–313]	144 [71–222]	91 [20–313]	0.4
Diuretics	120	43 (36)	9 (38)	34 (35)	0.8
Time on diuretics, days	35	3 [1–7]	3 [1–6]	3 [1–6]	0.7
Time from admission to diuretics	37	2 [0–3]	3 [0–3]	2 [0–3]	0.8
Antibiotics	123	69 (56)	17 (68)	52 (53)	0.2
Intravenous immunoglobulin	96	31 (32)	6 (33)	25 (32)	0.9
IVIG started before flare	127	24 (19)	5 (19)	19 (19)	0.9
IVIG started during flare	127	24 (19)	4 (15)	20 (20)	0.8
Corticosteroids	127	10 (8)	10 (38)	0 (0)	< 0.001
Complications in ICU					
Acute respiratory distress syndrome	122	9 (7)	5 (20)	4 (4)	0.02
Pulmonary edema^d^	116	20 (17)	3 (14)	17 (18)	0.8
Left ventricular dysfunction	81	21 (26)	4 (24)	17 (27)	> 0.99
Arrhythmia or conduction disorder	127	17 (13)	4 (15)	13 (13)	0.7
Compartment syndrome	124	32 (26)	8 (32)	24 (24)	0.4
Outcome					
Time in ICU, days	122	5 [2–9]	5 [2–9]	6 [1–11]	0.9
Day-28 mortality	127	27 (21)	11 (42)	17 (17)	0.005

MG-CLS; monoclonal gammopathy-associated capillary-leak syndrome; BMI, body-mass index; Ig, immunoglobulin; ICU, intensive care unit; SOFA, sequential organ failure assessment; APACHE, Acute Physiology and Chronic Health Evaluation II; CPK, creatine phosphokinase; IVIG, intravenous immunoglobulins. Continuous variables are expressed as mean (standard deviation) or median [interquartile range 25–75] and compared with Student’s t-test or Wilcoxon’s rank test; categorical variables are expressed as n (%) and compared with Fischer’s exact test. ICU admission values refer to the most abnormal measurements recorded within the first 24 h of the ICU stay

^a^Number of data available

^b^Addition of the total volumes of crystalloids, colloids, human albumin, and bicarbonates given during the ICU stay

^c^Calculated as: dobutamine (µg/kg/min) + 10 × milrinone (µg/kg/min) + dopamine (µg/kg/min) + 100 × epinephrine (mcg/kg/min) + 100 × norepinephrine (µg/kg/min) + 10,000 × vasopressin (µg/kg/min)

^d^Recovery-phase pulmonary edema

### Comparison according the SARS-CoV-2 status among the 127 episodes

**Fig. 2:**
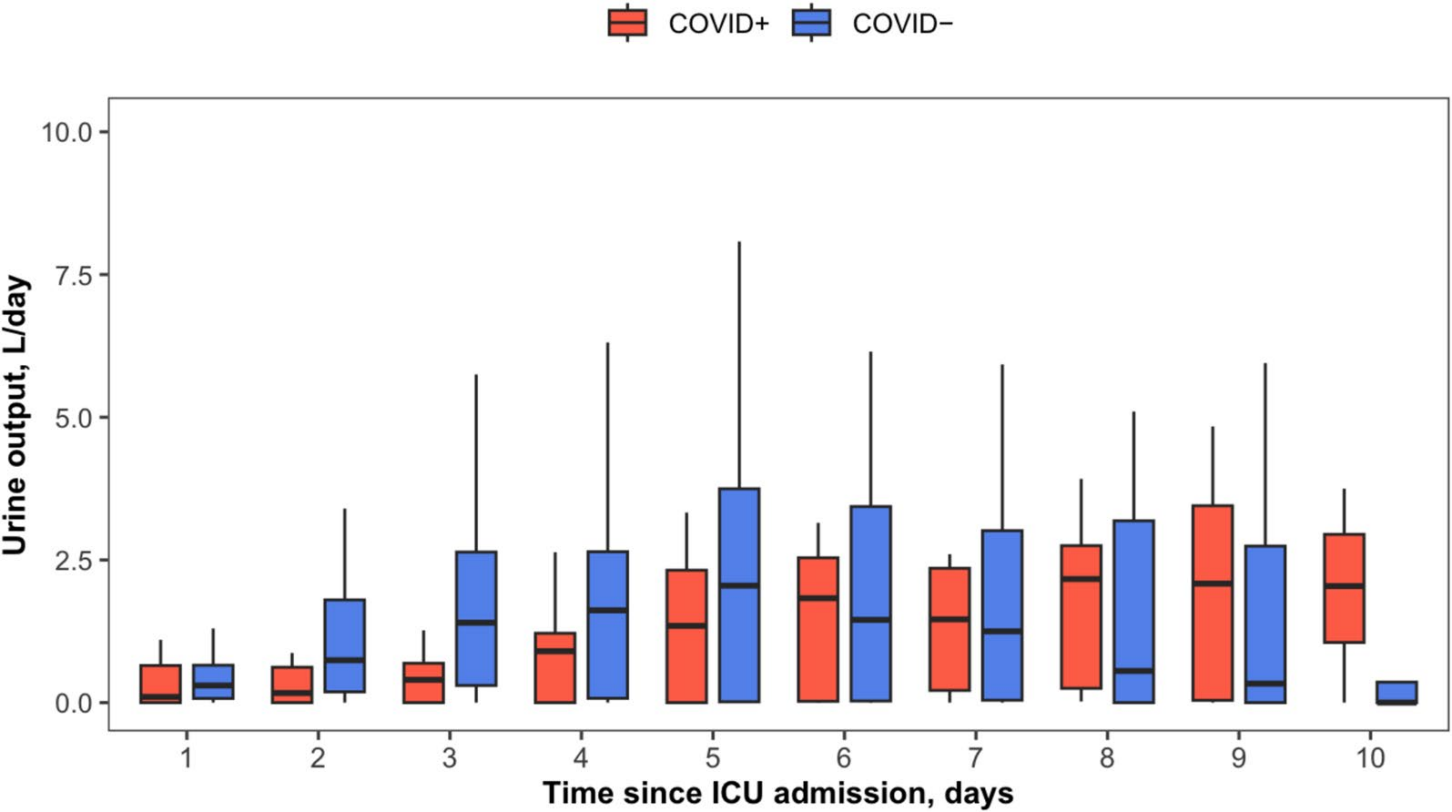
Day 1-to-10 In-ICU urine output with comparison by SARS-CoV-2 Status. ICU, intensive care unit; CLS, capillary-leak syndrome; COVID-19, coronavirus disease 2019. Box-plots values: internal horizontal line = median; lower and upper box limit = 25 th–75 th percentiles; whiskers = 0 th-100 th percentiles

**Fig. 3:**
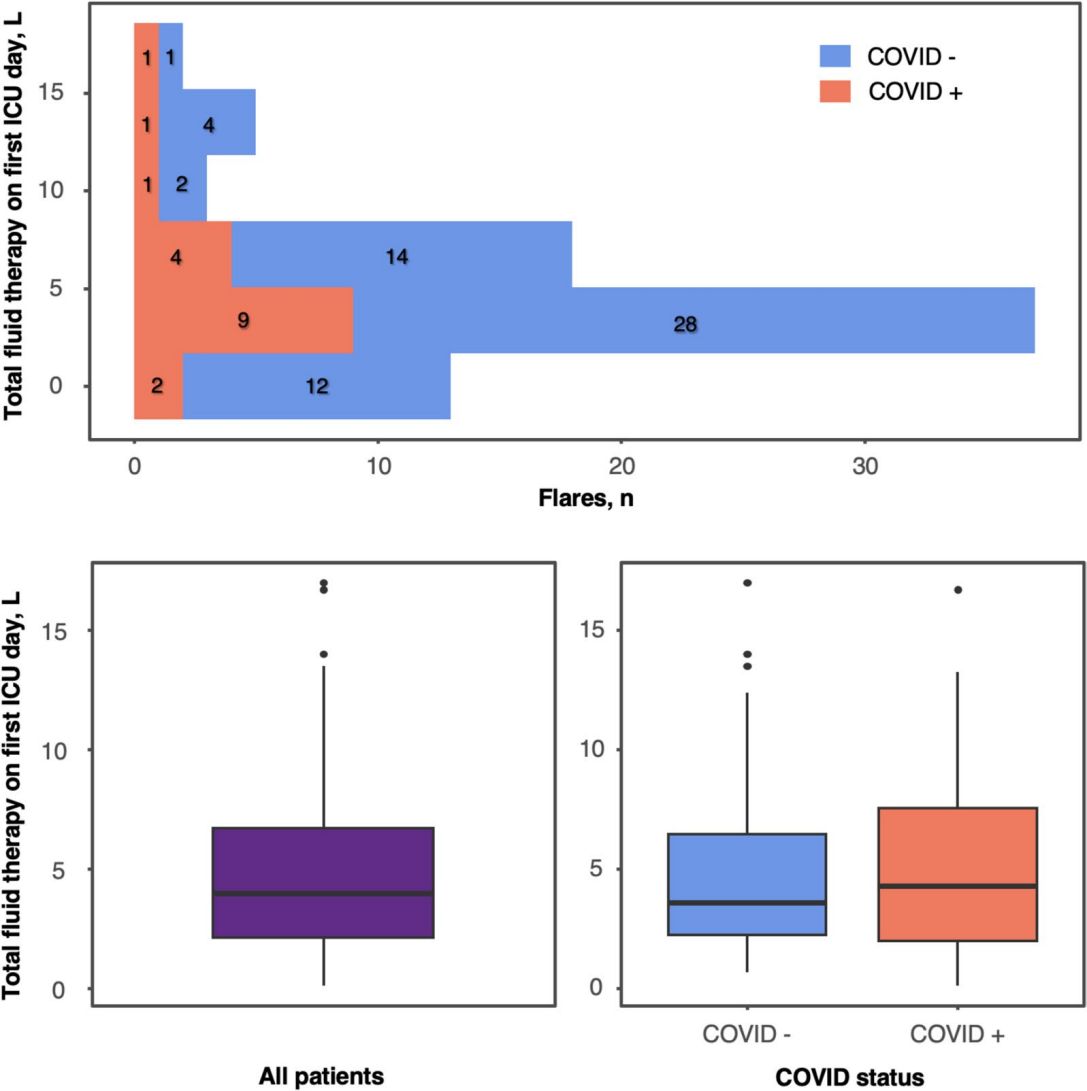
Cumulated fluid therapy on first ICU day with comparison by SARS-CoV-2 status. ICU, intensive care unit; COVID-19, coronavirus disease 2019. Box-plots values: internal horizontal line = median; lower and upper box limit = 25 th–75 th percentiles; whiskers = 0 th-100 th percentiles; black dots = outliers

No significant differences were observed between SARS-CoV-2 positive and negative episodes regarding reasons for ICU admission, SOFA and APACHE scores, weight, protein levels, creatinine levels, or arterial lactate levels (Table 2). Baseline hemoglobin levels tended to be higher in SARS-CoV-2 positive episodes (21.0 vs. 20.0 g/dL, p = 0.07), and CPK levels were significantly elevated (1,116 vs. 156 IU/L, p = 0.002) in these cases. The cumulative fluid therapy administered on day 1 did not differ significantly between the two groups, but maximum weight change in the ICU was greater in SARS-CoV-2 positive episodes (13 vs. 7 kg, p = 0.03). Figures 2 and 3 illustrate daily urine output and cumulative day-1 fluid therapy across all flares, stratified by SARS-CoV-2 status. SARS-CoV-2 positive episodes more frequently required mechanical ventilation (69% vs. 38%, p = 0.004) and renal replacement therapy (54% vs. 31%, p = 0.03), while the use of vasoactive drugs, diuretics, antibiotics, and IVIG was similar in both groups. Corticosteroids were only given in some SARS-CoV-2 positive episodes as they have never show to be effective in Clarkson disease severe episodes. Acute respiratory distress syndrome occurred more often in SARS-CoV-2 positive cases (20% vs. 4%, p = 0.02). Day-28 mortality was significantly higher in SARS-CoV-2 positive episodes compared to SARS-CoV-2 negative episodes (42% vs. 17%, p = 0.005). A detailed comparison stratified by SARS-CoV-2 status among single patients is provided in Supplemental Table S2.

### Mixed model univariable and multivariable analysis

**Fig. 4:**
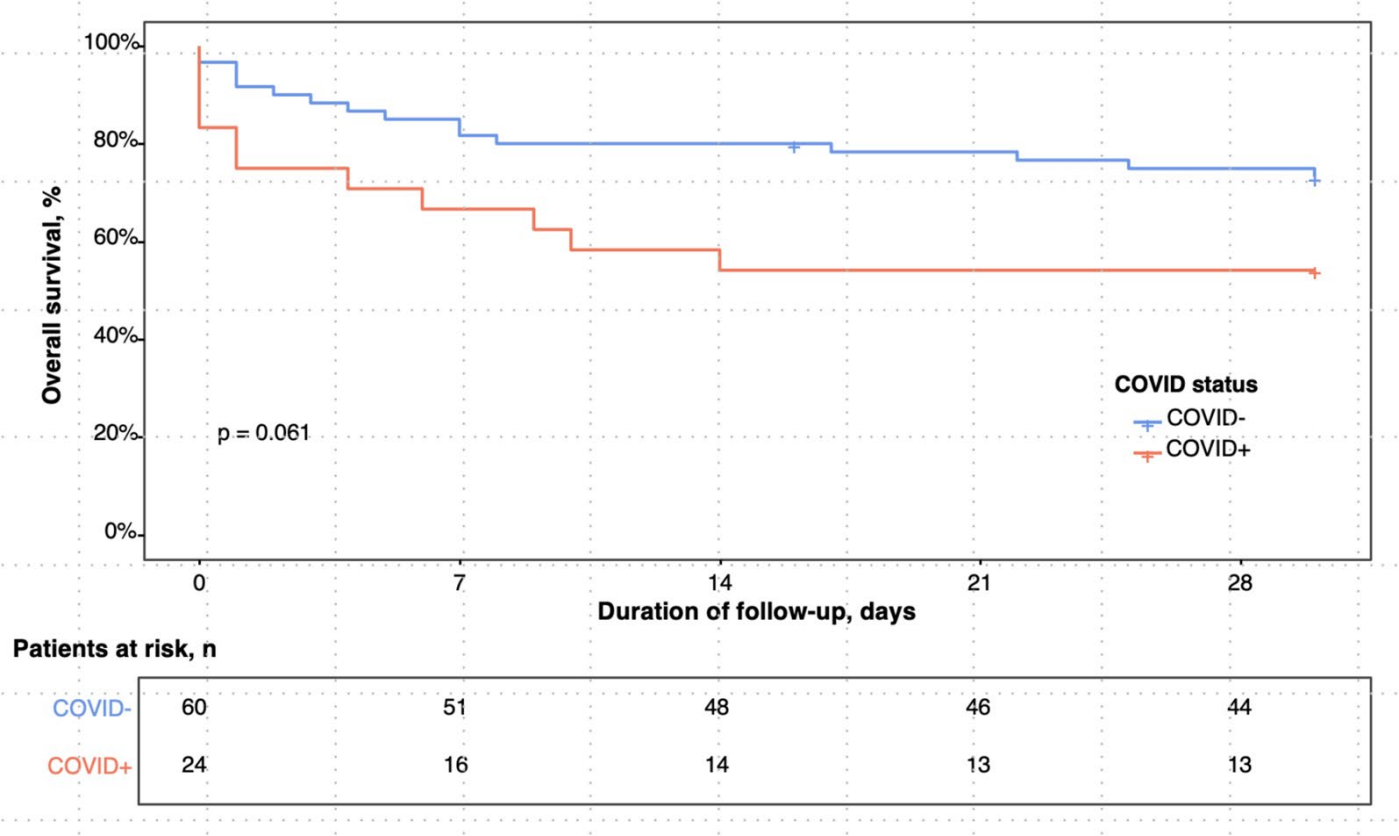
Kaplan–Meier Curves representation of the cumulative probability of survival until 28 days after ICU admission with comparison by COVID-19 status. ICU, intensive care unit; COVID-19, coronavirus disease 2019. Comparison According COVID-19 Status performed with the Log-Rank Test

The mixed model univariable analysis (Table 3) identified SARS-CoV-2 infection (OR 5.18 [1.19–22.5], p = 0.03) as significantly associated with 28-day mortality, alongside several variables measured at ICU admission: SOFA score (OR 15.2 [2.55–91.0], p = 0.003), APACHE II score (OR 1.11 [1.02–1.21], p = 0.01), albumin level (OR 0.93 [0.87–0.99], p = 0.02), and lactate level (OR 5.67 [1.98–16.2], p = 0.001). Other factors associated with 28-day mortality included day 1 fluid therapy volume (OR 1.25 [1.06–1.47], p = 0.008), mechanical ventilation (OR 38.4 [5.79–255], p < 0.001), renal replacement therapy (OR 517 [11.6–23,072], p = 0.001), and antibiotic use (OR 3.32 [1.10–10.0], p = 0.03). In the multivariable analysis, which excluded variables considered as mediators rather than confounders, only SARS-CoV-2 infection remained significantly linked to 28-day mortality: OR 4.67 [1.08–20.1], p = 0.04. The Kaplan–Meier curve (Fig. 4) illustrates the cumulative probability of survival from ICU admission to day 28, stratified by SARS-CoV-2 status. Notably, the use of IVIG did not demonstrate any association with improved 28-day survival.

**Table 3:** Univariable and multivariable mixed model of factors associated with 28-day mortality

Variables	Univariable analysis		Multivariable analysis	
	OR [95% CI]	p-value	OR [95% CI]	p-value
Age, years	1.03 [0.98 to 1.08]	0.2	1.02 [0.97 to 1.07]	0.4
Male	0.65 [0.24 to 1.75]	0.4		
BMI, kg/m^2^	1.00 [0.89 to 1.13]	0.9	1.01 [0.88 to 1.16]	0.9
Monoclonal gammopathy	0.75 [0.16 to 3.58]	0.7		
Date of flare				
≤ 2009	–			
2010–2019	0.50 [0.13 to 1.98]	0.3		
≥ 2020	2.05 [0.49 to 8.62]	0.3		
Identified trigger	1.06 [0.38 to 2.94]	0.9		
SARS-CoV-2 infection	5.18 [1.19 to 22.5]	0.03	4.67 [1.08 to 20.10]	0.04
SOFA score at ICU admission	15.2 [2.55 to 91.0]	0.003		
APACHE score at ICU admission	1.11 [1.02 to 1.21]	0.01		
Hemoglobin level at ICU admission, g/dL	1.01 [0.87 to 1.16]	0.9		
Albumin at ICU admission, g/L	0.93 [0.87 to 0.99]	0.02		
Lactate level at ICU admission, mmol/L	5.67 [1.98 to 16.2]	0.001		
Maximum weight change, kg	1.03 [0.98 to 1.09]	0.2		
Fluid therapy on day 1, L	1.25 [1.06 to 1.47]	0.008		
Fluid therapy > 10L on day 1	5.03 [1.06 to 23.8]	0.04		
Mechanical ventilation	38.4 [5.79 to 255]	< 0.001		
Renal replacement therapy	517 [11.6 to 23.07]	0.001		
IVIG before flare	0.12 [0.02 to 0.96]	0.05		
IVIG during flare	3.5 [0.88 to 14.00]	0.08		
Corticosteroids	2.9 [0.56 to 14.70]	0.2		
Diuretics	0.25 [0.06 to 1.15]	0.07		
Antibiotics	3.32 [1.10 to 10.0]	0.03		
Left ventricular dysfunction	2.22 [0.59 to 8.34]	0.2		
Arrhythmia or conduction disorder	3.74 [0.89 to 15.7]	0.07		

COVID-19; coronavirus disease 2019; BMI, body-mass index; ICU, intensive care unit; SOFA, sequential organ failure assessment; APACHE, Acute Physiology and Chronic Health Evaluation II. ICU admission values refer to the most abnormal measurements recorded within the first 24 h of the ICU stay

## Discussion

Clarkson disease, a rare monoclonal gammopathy of clinical significance, remains an area of considerable uncertainty with numerous unanswered questions and unmet therapeutic needs. The COVID-19 pandemic has brought renewed attention to the disease: a rising number of new cases have been diagnosed, COVID-19 vaccines have been implicated in inducing capillary leak episodes [15], many patients with previously stable disease have experienced relapses [8], and long-term survival has been adversely impacted by SARS-CoV-2 [9]. Viruses are recognized as significant triggers for MG-CLS episodes. Even in the post-pandemic era, SARS-CoV-2 infection continues to be associated with a substantial proportion of initial episodes and relapses. What remains unclear is whether the increased mortality observed among MG-CLS patients is primarily due to the surge in relapses or if COVID-19-triggered capillary leak episodes are inherently more severe than those caused by other triggers. Our study provides valuable insights into this question, as well as broader understanding of severe MG-CLS episodes.

First, we present the largest series of MG-CLS critically ill cases to date, encompassing patients from multiple institutions and countries. The sex ratio, patient age, frequency and type of monoclonal gammopathy, identified triggers, incidence of compartment syndrome, and early mortality rates are consistent with those reported in our previous studies [2]. Second, various parameters—including maximum hemoglobin, lactate, CPK, weight changes, minimum albumin levels, and the frequencies of mechanical ventilation and renal replacement therapy—highlight that SARS-CoV-2-positive episodes were more severe than those triggered by other factors. Notably, the mortality rate of SARS-CoV-2-related MG-CLS episodes was significantly higher than those associated with other triggers. Third, it is important to note that only a minority of SARS-CoV-2-positive episodes involved ARDS, suggesting that the severity of SARS-CoV-2 in these cases was independent of direct lung involvement. The incidence of ARDS in SARS-CoV-2-negative episodes was even lower, as the early phase of acute MG-CLS episodes is never associated with capillary-leak-related pulmonary edema. Fourth, fluid therapy remains the most frequent symptomatic treatment for severe acute episodes. However, due to the heterogeneity of the available data, we were unable to draw definitive conclusions regarding its impact on mortality or to recommend a specific type of solute. Lastly, our findings underscore the two primary complications of severe MG-CLS episodes: compartment syndrome and left ventricular dysfunction. These complications appear to be interconnected. Lower limb compartment syndrome arises from massive fluid resuscitation, skeletal muscle leakage, and the rigidity of the muscle compartments. Similarly, left ventricular dysfunction may be attributed to myocardial edema occurring within an inextensible pericardium, effectively representing a form of pericardial compartment syndrome.

Today, we favor the term *MG-CLS* over the earlier, more ambiguous designation"systemic capillary leak syndrome."However, in our series, some patients were included despite not having confirmed monoclonal gammopathy. Our findings are consistent with numerous studies published since the disease’s initial description, which report a 70% to 80% prevalence of monoclonal gammopathy [16–19]. In a prior series focused on the long-term follow-up of MG-CLS, we observed a 100% rate of monoclonal gammopathy, reflecting stricter inclusion criteria at that time. In this study, we opted to include a small subset of patients who exhibited the classic clinical features of Clarkson disease and had no alternative diagnosis, but who succumbed during their first episode, when monoclonal gammopathy is often either not found or not investigated thoroughly.

The strong association between COVID-19 and MG-CLS, as well as the increased mortality observed in SARS-CoV-2 positive episodes, remains poorly understood. The pathophysiology of MG-CLS, the role of monoclonal gammopathy, and the mechanism of action of IVIG during acute episodes are not yet fully elucidated [20]. Given that other viral and bacterial infections are established triggers for disease relapses, it is plausible that the hyperinflammatory state induced by COVID-19—characterized by elevated levels of pro-inflammatory cytokines such as IL-6, IL-1β, and TNF-α—might exacerbate endothelial dysfunction and enhance vascular permeability [21]. Furthermore, recent translational studies have demonstrated a specific tropism of SARS-CoV-2 for the endothelium, potentially amplifying endothelial dysfunction and contributing to the pathogenesis of vascular leak [22, 23, 24]. However, the observation that SARS-CoV-2 vaccines can also trigger severe MG-CLS episodes suggests that a specific viral protein, rather than the viral infection itself, may play a role in favoring MG-CLS episodes.

IVIG remains the cornerstone treatment for preventing relapses and improving long-term survival in MG-CLS [5, 9, 11, 25]. The mechanism of action of IVIG in this condition remains unknown. However, its role during acute severe episodes of MG-CLS is more contentious. While some small series have suggested that IVIG may reverse ongoing severe episodes [26, 27], our previous research did not find an association between IVIG use and improved survival outcomes [2]. Furthermore, the high cost of IVIG, recent supply shortages [28, 29], and reimbursement challenges for this indication highlight the need for its careful and judicious use. Notably, in the present study, we found no evidence of a survival benefit associated with IVIG administration during the acute phase of MG-CLS, further questioning its routine application in this context.

This study has several limitations that merit discussion. First, its retrospective design inherently limits its external validity. Second, although this is the largest series of severe MG-CLS episodes requiring ICU admission to date, the sample size remains small and a significant number of data are missing, which reduces the statistical power of the study, especially when considering the width of the confidence interval associated with the odds ratio (OR) for 28-day mortality in relation to SARS-CoV-2 infection A larger international cohort is needed to refine the effect estimate. Third, management practices may have varied across the inclusion period and participating institutions, particularly between SARS-CoV-2 positive and negative episodes. However, this variability mirrors the real-world management of severe MG-CLS. Fourth, when comparing patient characteristics and outcomes by SARS-CoV-2 status, we selected the most recent flare for patients with multiple episodes, an arbitrary decision that may introduce selection bias. To mitigate this, we employed a mixed-effects model that accounted for repeated measures to identify factors associated with mortality. Finally, all patients from our previous cohort were included in this study, contributing to the reproducibility of data across both series.

## Conclusion

In this large cohort of MG-CLS episodes requiring ICU admission, SARS-CoV-2 infection as a trigger was associated with significantly higher 28-day mortality compared to other triggers. Further research is needed to elucidate the specific effects of SARS-CoV-2on the outcomes of MG-CLS.

## Supplementary Information

Additional file 1.

## Data Availability

None.

## Abbreviations

APACHE II: Acute Physiology and Chronic Health Disease Classification System II
ARDS: Acute Respiratory Distress Syndrome
COVID-19: Coronavirus Disease 2019
ICU: Intensive Care Unit
IVIG: Intravenous immunoglobulins
MG-CLS: Monoclonal Gammopathy-Associated Capillary Leak Syndrome
SOFA: Sequential Organ Failure Assessment
SARS-CoV-2: Severe Acute Respiratory Syndrome Coronavirus 2
