# Supplementary material for: Characteristics of SARS-CoV-2-associated severe episodes of monoclonal gammopathy-associated capillary leak syndrome (Clarkson disease)
Source: Ann Intensive Care. 2025 May 26;15:72. doi: 10.1186/s13613-025-01483-7 (PMC12106253; doi:10.1186/s13613-025-01483-7)
Supplement: Supplementary file 1 — Additional file 1. [file 13613_2025_1483_MOESM1_ESM.docx]

| **Supplementary Table 1. Missing data analysis of variables used in models** | | | | | | |
| --- | --- | --- | --- | --- | --- | --- |
| Variable | COVID- n_missing | COVID- Missing, % | COVID+ n_missing | COVID+ Missing, % | Overall n_missing | Overall Missing % |
| ICU admission – diuretics start delay, days | 71 | 70.3 | 19 | 73.1 | 90 | 70.9 |
| Maximum weight change | 35 | 34.7 | 13 | 50 | 48 | 37.8 |
| Albumin at ICU, g/L | 28 | 27.7 | 12 | 46.2 | 40 | 31.5 |
| APACHE score at ICU | 33 | 32.7 | 11 | 42.3 | 44 | 34.6 |
| Change in hemoglobin level | 14 | 13.9 | 9 | 34.6 | 23 | 18.1 |
| Left Ventricular dysfunction | 37 | 36.6 | 9 | 34.6 | 46 | 36.2 |
| Infused fluids on day 1, L | 41 | 40.6 | 8 | 30.8 | 49 | 38.6 |
| Infused fluids > 10L on day 1 | 41 | 40.6 | 8 | 30.8 | 49 | 38.6 |
| Body mass index | 29 | 28.7 | 7 | 26.9 | 36 | 28.3 |
| Intravenous Immunoglobulin | 21 | 20.8 | 6 | 23.1 | 27 | 21.3 |
| Response to fluid challenge | 15 | 14.9 | 5 | 19.2 | 20 | 15.7 |
| Lactate level at ICU | 28 | 27.7 | 5 | 19.2 | 33 | 26 |
| Total SOFA score at ICU | 7 | 6.9 | 4 | 15.4 | 11 | 8.7 |
| Hemoglobin level at ICU | 1 | 1 | 1 | 3.8 | 2 | 1.6 |
| Antibiotics | 3 | 3 | 1 | 3.8 | 4 | 3.1 |
| COVID infection | 0 | 0 | 0 | 0 | 0 | 0 |
| Age, years | 0 | 0 | 0 | 0 | 0 | 0 |
| Male sex | 0 | 0 | 0 | 0 | 0 | 0 |
| Date of flare | 0 | 0 | 0 | 0 | 0 | 0 |
| Monoclonal gammopathy | 0 | 0 | 0 | 0 | 0 | 0 |
| Trigger of flare | 0 | 0 | 0 | 0 | 0 | 0 |
| Arrhythmia or conduction disorder | 0 | 0 | 0 | 0 | 0 | 0 |
| Mechanical ventilation | 3 | 3 | 0 | 0 | 3 | 2.4 |
| Renal replacement therapy | 0 | 0 | 0 | 0 | 0 | 0 |
| IVIG started before flare | 0 | 0 | 0 | 0 | 0 | 0 |
| IVIG started during flare | 0 | 0 | 0 | 0 | 0 | 0 |
| Abbreviations: Ig, immunoglobulin; ICU, intensive care unit; SOFA, sequential organ failure assessment; APACHE, Acute Physiology and Chronic Health Evaluation II; IVIG, intravenous immunoglobulins. | | | | | | |

| **Supplemental Table 2. Characteristics, Treatments and Outcomes Of Each MG-CLS Patients Last Flare With Comparison According SARS-CoV-2 Status** | | | | |
| --- | --- | --- | --- | --- |
| **Variables** | **n^a^** | **SARS-CoV-2+**  **n=24** | **SARS-CoV-2-**  **n=60** | **p-value** |
| BMI, kg/m^2^ | 55 | 24.6 [23.1-28.8] | 25.6 [23.1-28.1] | 0.9 |
| Weight, kg | 59 | 72 [63-84] | 80 [69-84] | 0.5 |
| Monoclonal gammopathy | 84 | 19 (79) | 55 (92) | 0.1 |
| Cause of ICU admission | 84 |  |  | 0.1 |
| Hypovolemic shock |  | 22 (92) | 49 (82) |  |
| Cardiogenic shock |  | 0 (0) | 6 (10) |  |
| Coma |  | 1 (4) | 2 (3) |  |
| Respiratory distress |  | 0 (0) | 3 (5) |  |
| Cardiac arrest |  | 1 (4) | 0 (0) |  |
| Acute kidney insufficiency |  | 0 (0) | 0 (0) |  |
| Identified trigger | 84 | 24 (100) | 31 (52) | <0.001 |
| Type of trigger | 84 |  |  | <0.001 |
| Confirmed infection |  | 24 (100) | 6 (10) |  |
| Suspected infection |  | 0 (0) | 25 (42) |  |
| Hormonal |  | 0 (0) | 1 (2) |  |
| None |  | 0 (0) | 28 (47) |  |
| Clinical and laboratory findings at ICU admission |  |  |  |  |
| SOFA score at ICU admission | 76 | 5 [3-11] | 5 [3-10] | 0.5 |
| APACHE score at ICU | 49 | 22 [18-37] | 21 [14-29] | 0.3 |
| Weight at ICU admission, kg | 59 | 79 [69-93] | 82 [70-90] | 0.9 |
| Weight change at ICU admission, kg | 50 | 8 [3-15] | 1 [0-7] | 0.06 |
| Maximum weight change in ICU, kg | 48 | 13 [10-20] | 7 [2-17] | 0.1 |
| Hemoglobin level at ICU admission, g/dL | 82 | 21.3 [19.8-22.6] | 20.0 [17.9-21.9] | 0.08 |
| Maximal change in hemoglobin in ICU, g/dL | 65 | 11.0 [5.7-12.7] | 10.2 [7.7-13.6] | 0.6 |
| Protidemia at ICU admission, g/L | 62 | 46 [41-69] | 53 [38-60] | 0.8 |
| Minimal protidemia in ICU, g/L | 52 | 41 [21-48] | 38 [26-52] | 0.7 |
| Albumin at ICU admission, g/L | 60 | 21 [15-30] | 28 [18-30] | 0.3 |
| Minimal albumin in ICU, g/L | 52 | 16 [14-24] | 23 [18-28] | 0.07 |
| Creatinine level at ICU admission, µmol/L | 81 | 155 [111-235] | 148 [109-217] | 0.8 |
| Maximal creatinine in ICU, µmol/L | 70 | 223 [155-284] | 217 [130-258] | 0.5 |
| CPK level at ICU admission, UI/L | 57 | 1,115 [266-2,547] | 164 [105-1,104] | 0.02 |
| Maximal CPK in ICU, UI/L | 55 | 8,388 [927-12,024] | 1,196 [141-41,853] | 0.3 |
| Arterial lactate level at ICU admission, mmol/L | 64 | 8.0 [3.5-9.9] | 4.3 [2.4-7.0] | 0.1 |
| Maximal lactate level in ICU, mmol | 59 | 10.7 [7.8-14.0] | 5.7 [3.0-10.4] | 0.03 |
| Bicarbonate level at ICU admission, mmol/L | 70 | 12.0 [8.0-16.0] | 13.0 [10.6-15.6] | 0.5 |
| In-ICU treatments |  |  |  |  |
| Fluid therapy^b^ |  |  |  |  |
| Fluid therapy on day 1, L | 54 | 4.3 [2.0-7.3] | 3.0 [1.6-6.0] | 0.3 |
| Fluid therapy > 10L on day 1 | 54 | 2 (13) | 4 (11) | >0.99 |
| Mechanical ventilation | 83 | 18 (75) | 26 (44) | 0.01 |
| Renal replacement therapy | 84 | 14 (58) | 23 (38) | 0.1 |
| Time on renal replacement therapy, days | 34 | 2 [1-8] | 5 [2-16] | 0.3 |
| Vasoactive-inotropic score^c^ | 31 | 144 [103-308] | 96 [25-631] | 0.7 |
| Diuretics | 79 | 8 (36) | 18 (32) | 0.7 |
| Time on diuretics, days | 21 | 2 [0-6] | 3 [2-6] | 0.6 |
| Antibiotics | 81 | 16 (70) | 31 (53) | 0.2 |
| IVIG before flare | 84 | 4 (17) | 9 (15) | 0.9 |
| IVIG during flare | 84 | 4 (17) | 18 (30) | 0.2 |
| Corticosteroids | 84 | 10 (42) | 0 (0) | <0.001 |
| Complications in ICU |  |  |  |  |
| Acute respiratory distress syndrome | 80 | 5 (22) | 2 (3.5) | 0.02 |
| Pulmonary edema^d^ | 76 | 3 (15) | 11 (20) | 0.7 |
| Left ventricular dysfunction | 54 | 4 (27) | 12 (31) | >0.99 |
| Arrhythmia or conduction disorder | 84 | 3 (13) | 11 (18) | 0.7 |
| Compartment syndrome | 83 | 8 (35) | 15 (25) | 0.4 |
| Outcome |  |  |  |  |
| Day-28 mortality | 84 | 11 (46) | 16 (27) | 0.09 |
| Abbreviations: MG-CLS; monoclonal gammopathy-associated capillary-leak syndrome; BMI, body-mass index; Ig, immunoglobulin; ICU, intensive care unit; SOFA, sequential organ failure assessment; APACHE, Acute Physiology and Chronic Health Evaluation II; CPK, creatine phosphokinase; IVIG, intravenous immunoglobulins. Continuous variables are expressed as mean (standard deviation) or median [interquartile range 25-75] and compared with Student’s t-test or Wilcoxon’s rank test; categorical variables are expressed as n (%) and compared with Fischer’s exact test. ICU admission values refer to the most abnormal measurements recorded within the first 24 hours of the ICU stay.  **^a^**Number of data available.  ^b^Addition of the total volumes of crystalloids, colloids, human albumin, and bicarbonates given during the ICU stay.  ^c^Calculated as: dobutamine (µg/kg/min) + 10 x milrinone (µg/kg/min) + dopamine (µg/kg/min) + 100 x epinephrine (mcg/kg/min) +100 x norepinephrine (µg/kg/min) + 10,000 x vasopressin (µg/kg/min).  ^d^Recovery-phase pulmonary edema. | | | | |
